# Prognostic factors to predict the survival in patients with advanced gastric cancer who receive later‐line nivolumab monotherapy—The Asahikawa Gastric Cancer Cohort Study (AGCC)

**DOI:** 10.1002/cam4.4461

**Published:** 2021-11-29

**Authors:** Kazuyuki Tanaka, Hiroki Tanabe, Hiroki Sato, Chisato Ishikawa, Mitsuru Goto, Naoyuki Yanagida, Hiromitsu Akabane, Shiro Yokohama, Kimiharu Hasegawa, Yohei Kitano, Yuya Sugiyama, Kyoko Uehara, Yu Kobayashi, Yuki Murakami, Takehito Kunogi, Takahiro Sasaki, Keitaro Takahashi, Katsuyoshi Ando, Nobuhiro Ueno, Shin Kashima, Kentaro Moriichi, Keisuke Sato, Sayaka Yuzawa, Mishie Tanino, Masaki Taruiishi, Yasuo Sumi, Yusuke Mizukami, Mikihiro Fujiya, Toshikatsu Okumura

**Affiliations:** ^1^ Department of Gastroenterology Asahikawa Kosei Hospital Asahikawa Hokkaido Japan; ^2^ Division of Metabolism and Biosystemic Science, Gastroenterology and Hematology/Oncology Department of Medicine Asahikawa Medical University Asahikawa Hokkaido Japan; ^3^ Department of Gastroenterology Japanese Red Cross Asahikawa Hospital Asahikawa Hokkaido Japan; ^4^ Department of Surgery Asahikawa Kosei Hospital Asahikawa Hokkaido Japan; ^5^ Department of Gastroenterology Asahikawa Medical Center Asahikawa Hokkaido Japan; ^6^ Division of Gastrointestinal Surgery Asahikawa Medical University Asahikawa Hokkaido Japan; ^7^ Department of Pathology Asahikawa Kosei Hospital Asahikawa Hokkaido Japan; ^8^ Department of Diagnostic Pathology Asahikawa Medical University Hospital Asahikawa Medical University Asahikawa Hokkaido Japan; ^9^ Department of Gastroenterology Asahikawa City Hospital Asahikawa Hokkaido Japan

**Keywords:** biomarker, chemotherapy, gastric carcinoma, microsatellite instability, nomogram

## Abstract

**Background:**

Chemotherapy for advanced gastric cancer is recommended in the guidelines; however, later‐line treatment remains controversial. Since immune checkpoint inhibitors have been used for the treatment of various malignancies, trials have been performed for gastric cancer. A phase 3 trial indicated the survival benefit of nivolumab monotherapy for gastric cancer patients treated with prior chemotherapy regimens.

**Patients and methods:**

A regional cohort study was undertaken to determine the real‐world data of nivolumab treatment for patients with advanced or recurrent gastric cancer. The patients were enrolled for 2 years from October 2017 to October 2019 and were prospectively followed for 1 year to examine the overall survival (OS). The patient characteristics were analyzed in a multivariate analysis and a nomogram to predict the probability of survival was generated.

**Results:**

In total, 70 patients who received nivolumab as ≥third‐line chemotherapy were included in the Asahikawa Gastric Cancer Cohort. The median OS was 7.5 (95% CI, 4.8–10.2) months and the response rate was 18.6%. Diffuse type classification, bone metastasis, high neutrophil/lymphocyte ratio, and high CRP were associated with poor OS/prognosis in the multivariate analysis. A nomogram was developed based on these clinical parameters and the concordance index was 0.80 (95% CI, 0.68–0.91). The responders were aged and were frequently diagnosed with intestinal type gastric cancer, including patients with a HER2‐positive status (27.3%) or microsatellite instability‐high (27.3%) status.

**Conclusions:**

The regional cohort study of nivolumab monotherapy for gastric cancer patients revealed prognostic factors and a nomogram was developed that could predict the probability of survival.

## Graphical abstract

Development and validation of the nomogram. The nomogram of different risk factors for survival at 7.5 months in gastric cancer patients treated with nivolumab.

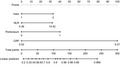

## 1 INTRODUCTION

Nivolumab, an immune checkpoint inhibitor, has been investigated in the treatment of unresectable or recurrent chemotherapy‐resistant gastric cancer. A phase 3 trial (ATTRACTION‐2) indicated the survival benefits of nivolumab for the Asian patients with gastric and gastroesophageal junction cancer that was treated with prior chemotherapy regimens.^1^ The study reported a median overall survival (OS) of 5.26 months with nivolumab and 4.14 months with placebo. Nivolumab treatment was then recommended for gastric cancer patients as ≥third‐line chemotherapy in the gastric cancer treatment guidelines of Asian countries.^2^, ^3^ However, nivolumab treatment has only been approved as later‐phase chemotherapy for gastric cancer in very limited countries.^4^ As the European Society of Medical Oncology clinical practice guidelines for the diagnosis, treatment, and follow‐up of gastric cancer do not recommend any third‐line treatment, the effectiveness of later‐line chemotherapy for advanced gastric cancer is still controversial.^5^, ^6^

A total of 782,685 gastric cancer‐related deaths were reported worldwide in 2018.^7^ The gastric cancer incidence reached 1,033,701 and the disease was frequently found in Eastern Asia, Eastern Europe, South America, Western Asia, and other countries. Gastric cancer mortality in Japan is reported to be 30,000 patients/year,^8^ while the gastric cancer mortality in Asahikawa city, Hokkaido—which has a population of 350 million—is proximately 140 patients/year. We conducted a regional cohort study to assess the real‐world evidence in the survival data of gastric cancer patients who received nivolumab. The clinical information about the chemotherapy regimens that the patients received and their total survivals were also collected in a data center. We herein present the 1‐year follow‐up results of the Asahikawa gastric cancer cohort. The clinical characteristics were analyzed, and factors predicting the survival were investigated in the cohort group. The total survival period from the beginning of the chemotherapy to the death was additionally examined to investigate the impact of recent advances in gastric cancer treatment in a real‐world setting.

## 2 MATERIALS AND METHODS

### 2.1 Study design and patients

We performed a multicenter prospective cohort study of gastric carcinoma treated with nivolumab to obtain real‐world data. The Asahikawa gastric cancer cohort study group includes a special function hospital and four cancer medical cooperation base hospitals—where nivolumab treatment is conducted—in Asahikawa city, Hokkaido, Japan. All clinical data of gastric cancer patients who received nivolumab treatment were collected, and a data analysis was performed at the section of Cancer Genomic Medicine in Asahikawa Medical University. The protocol was approved by the Asahikawa Medical University Research Ethics Committee (approval #1912) and by each institute. Patients were enrolled for 2 years from October 2017 to October 2019. Patients of ≥20 years of age with unresectable advanced or recurrent gastric cancer that was confirmed to be adenocarcinoma were eligible for inclusion in this study. Patients treated with standard chemotherapy regimens and patients with carcinomas that were refractory to or intolerant of the chemotherapy. A 1‐year follow‐up period after the end of the enrollment period was assigned, and collection of survival data was ended on October 2020. Clinical data, including the sex, age, tumor status (macroscopic type, histological classification, HER2 status, and site of metastasis), history of gastrectomy, previous chemotherapy, and pretreatment blood chemistry data (neutrophil/lymphocyte ratio [NLR], C‐reactive protein [CRP], and albumin level). Any case with immunohistochemistry (IHC) 3+ or IHC 2+ with fluorescence in situ hybridization (FISH) + was considered to be HER2‐positive according to the Pharmaceutical and Medical Devices Agency and the European Medicines Agency. The NLR at the time of the first administration of nivolumab was defined high and low (cut‐off value: 5).^9^ The Glasgow prognostic score (GPS) at the first administration was defined according to a previous report.^10^ Patients with an elevated CRP (>1.0 mg/dl) and low albumin level (<3.5 g/dl) were assigned a score of 2. Either one of them was assigned a score of 1; no factors were assigned a score of 0.

The patients received 3 mg/kg or 240 mg/body nivolumab intravenously every 2 weeks. The patients received treatment until disease progression or until the development of unacceptable toxicity. Tumor responses were assessed by the investigator using computed tomography (CT) or magnetic resonance imaging (MRI), according to the Response Evaluation Criteria in Solid Tumors guidelines. Tumor assessment was performed by each investigator and after the end of the treatment. Patients with a complete response (CR) or partial response (PR) were classified as responders. The disease control rate was defined as the proportion of patients with CR, PR, or stable disease (SD). Adverse events (AEs) were evaluated by the National Cancer Institute Common Terminology Criteria for Adverse Events (CTCAE) version 4.0. The incidence of grade >1 treatment‐related AEs (TRAEs) was evaluated in this study.

### 2.2 Outcomes

The primary endpoint was overall survival (OS), which was defined as the period from the initiation of nivolumab treatment to the death of the patients. The secondary endpoints were total OS, defined as the period from the beginning of the primary chemotherapy to death, the treatment continuation rate, and the objective response rate (proportion of responders). We also performed an exploratory post hoc analysis of OS according to response and existence of TRAEs.

### 2.3 Statistical analysis

The median OS and 95% confidence interval (CI) were calculated using the Kaplan–Meier method. The log‐rank test was used to compare the difference in OS between the groups. Hazard ratios (HRs) with 95% CIs were calculated using a stratified Cox proportional hazards model. The prognostic variables were assessed in a multivariable analysis. The histological type, liver metastasis, peritoneal metastasis, and CRP were selected as explanatory variables, as their *p* values in the univariable analysis were significant (*p* ≤ 0.001). The NLR was selected as a variable based on a retrospective observational study.^9^ All analyses were performed using the SPSS software program, version 25 (IBM, New York, NY, USA), and *p* values of <0.05 were considered to indicate statistical significance.

### 2.4 Construction and validation of a nomogram

Independent risk factors associated with longer survival were chosen to construct a nomogram using R version 4.0.3 software (The R Foundation for Statistical Computing, Vienna, Austria). The nomogram was generated by the Bell Curve for Excel (Social Survey Research Information Co. Ltd., Tokyo, Japan). The probability of survival at the time of the mean OS was predicted from the nomogram. Validation of the nomogram was performed through repeated independent samplings based on our cohort. The concordance index (C‐index) provided a probability value between the observed and predicted probability. A receiver operator characteristics (ROC) curve was created using the nomogram and independent risk factors.

### 2.5 Histochemical analysis

Responders were subjected to a histological analysis after obtaining their written informed consent. Formalin‐fixed paraffin‐embedded (FFPE) surgically resected specimens and endoscopically biopsied tissue were used for the histological and immunohistochemical examinations. Mismatch repair (MMR) protein expression was evaluated with IHC. MMR proteins were assessed in the tumor cells with antibodies against mutL homolog 1 (MLH1), postmeiotic segregation increase 2 (PMS2), MutS homolog 2 (MSH2), and MutS homolog 6 (MSH6). Cancer with the decreased expression levels was defined as MMR‐deficient (dMMR).

## 3 RESULTS

### 3.1 Patient characteristics and treatment efficacy

During the patient enrollment period, 70 gastric cancer patients received nivolumab treatment. The patients’ characteristics are shown in Table 1. The median age was 69 (39–84) years, 65.7% were male, and 62.3% were elderly (≥65). All patients were histologically diagnosed with gastric adenocarcinoma, with an unresectable tumor and/or recurrent metastatic cancer. HER2 (evaluated by IHC and/or FISH) was positive in 12 patients (17.1%). The median age at the first diagnosis was 66 (36–83) years and all patients had previously received chemotherapy. The previous chemotherapy regimens are listed in Table S1 and S2. Twenty‐six patients (37.1%) received >2 regimens before nivolumab treatment.

**TABLE 1:** Patient characteristics

	Number of patients (%)
Gender	
Male	46 (65.7)
Female	24 (34.3)
Age	
Median (range)	69 (39–84)
Macroscopic type	
0	4 (5.7)
1	21 (30.0)
2	30 (42.9)
3	13 (18.6)
4	2 (2.9)
Histological classification	
Intestinal	36 (51.4)
Diffuse	34 (48.6)
HER2 status	
Positive	12 (17.1)
Negative	58 (82.9)
Metastatic site	
Liver	21 (30.0)
Lymph node	28 (41.4)
Bone	3 (4.3)
Lung	4 (5.7)
Peritoneum	34 (48.6)
Other organ	4 (5.7)
History of gastrectomy	31 (44.3)
Previous chemotherapy	
1,2	44 (62.9)
≥3	26 (37.1)
Pretreatment NLR	
Low	17 (24.3)
High	51 (72.9)
Pretreatment CRP (mg/dl)	
Median (range)	0.37 (0.0–9.4)
Pretreatment albumin (g/dl)	
Median (range)	3.3 (2.2–4.5)
Glasgow prognostic score	
0	21 (30.0)
1	28 (40.0)
2	15 (21.4)

Abbreviation: NLR, neutrophil/lymphocyte ratio.

**FIGURE 1:**
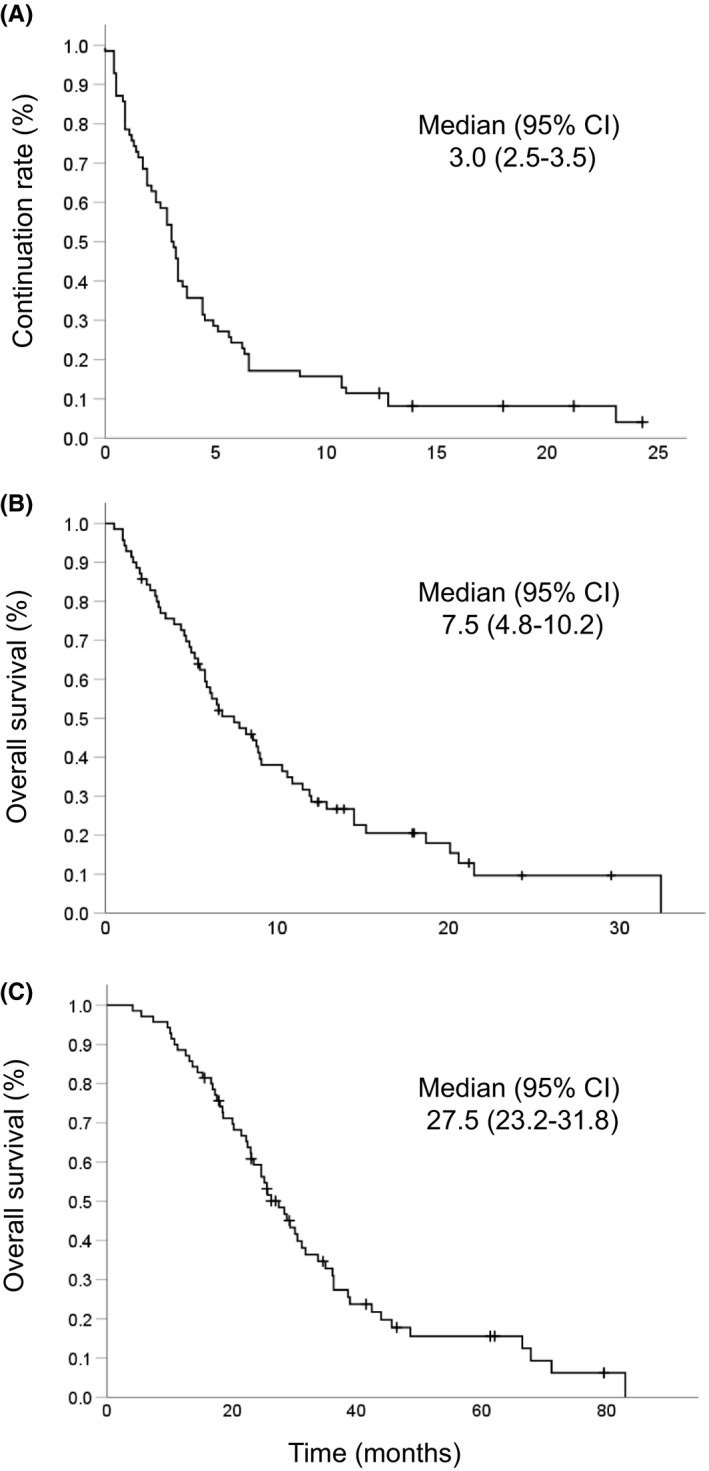
The Kaplan–Meier plots of continuation rate of nivolumab (A), overall survival (B) after 1 year of follow‐up. The Kaplan–Meier plot of total overall survival from the initiation of the primary treatment (C)

**FIGURE 2:**
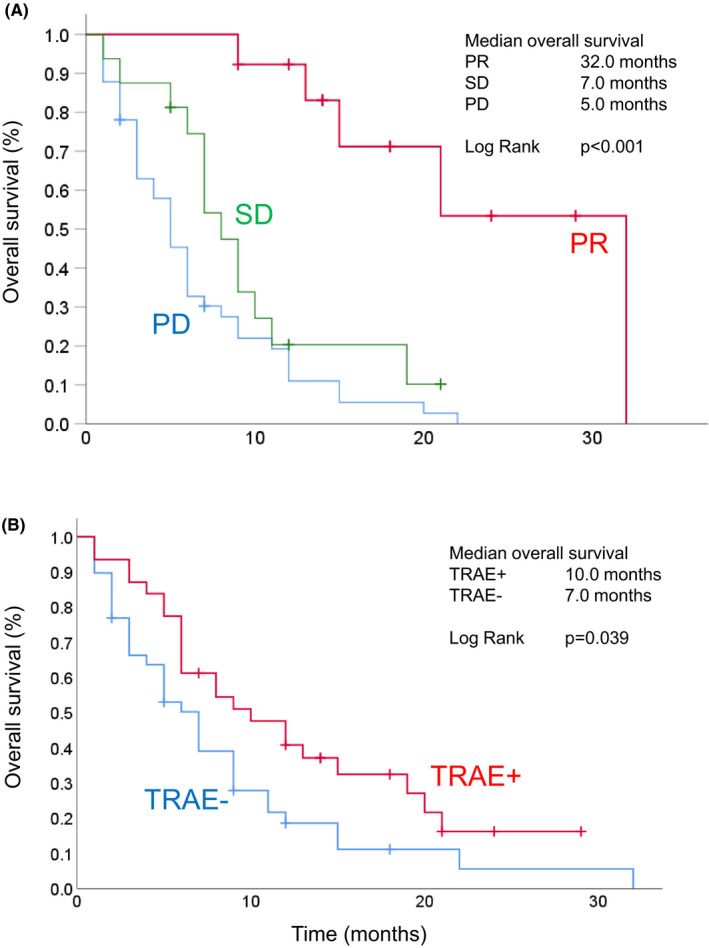
The Kaplan–Meier plots of sub‐analysis. (A) The sub‐analysis of overall survival according to the best overall response among patients. PR, partial response; SD, stable disease; PD, progressive disease. (B) The sub‐analysis of overall survival according to treatment‐related adverse events (AEs) among patients with and without AEs

The final analysis was performed 12 months after the end of the enrollment period. At this time point, 57 patients (81.4%) had died. Among 13 surviving patients (18.6%), 5 patients (7.1%) continued to receive nivolumab treatment. Tumor response could be evaluated with CT or MRI in all cases. The response rate was 18.6% (PR, *n* = 13; CR, *n* = 0) and the disease control rate was 41.4% (Table 2). The median duration of treatment was 3.0 months with six cycles of nivolumab (Figure 1A). Subsequent chemotherapy after the discontinuation of nivolumab was administered to 26 patients (37.1%). The regimens used are shown in Table S3. As the primary endpoint, the median OS reached 7.5 (95% CI, 4.8–10.2) months (Figure 1B). In this clinical cohort, the median total survival period from the beginning of the first‐line chemotherapy was 27.5 (95% CI, 23.2–31.8) months (Figure 1C). A subgroup analysis of OS according to the response was performed using the Kaplan–Meier curves (Figure 2A). Responders (patients with PR) showed longer median OS (32.0 months, 8.0, and 5.0 for PR, SD, and PD, respectively, *p *< 0.001).

**TABLE 2:** Best response to nivolumab treatment

		Number of patients (%)		
Response		Total	With TRAE	Without TRAE
Complete response	(CR)	0 (0)	0 (0)	0 (0)
Partial response	(PR)	13 (18.6)	8 (27.6)	5 (12.2)
Stable disease	(SD)	16 (22.9)	7 (24.1)	9 (22.0)
Progressive disease	(PD)	41 (58.6)	14 (48.3)	27 (65.9)
Disease control	(CR, PR, or SD)	29 (41.4)	15 (51.7)	14 (34.1)

Abbreviation: TRAE, treatment‐related adverse event.

### 3.2 Safety and efficacy

Overall, the incidence of TRAE in patients who received nivolumab was 41.4% (Table 2 and Table S3). Fatigue and liver functional disorder were frequently observed; however, there were no cases in which discontinuation or treatment delay was required due to common TRAEs. Out of the 29 patients with TRAEs, 18 cases were injured with severe TRAEs (grade ≥3) with specific AEs are shown in Table S3. The TRAEs included interstitial pneumonia, hypoadrenocorticism, hypothyroidism, colitis, and optic nerve disorder.

The response to nivolumab is shown in Table 2, and the response rate was the same as the PR rate due to the absence of CR cases. The response rate and disease control rate in patients with TRAEs were 27.6% (8/29) and 51.7% (15/29), respectively, whereas those in patients without TRAEs were 12.2% (5/41) and 34.1% (14/41). Patients with TRAEs did not show a significantly higher response in comparison to those without TRAEs (*p *= 0.103, chi‐squared test). The association between TRAEs and OS was analyzed with a log‐rank test and the Kaplan–Meier curves were determined (Figure 2B). The median OS in patients with and without TRAE was 10.0 (95% CI, 4.8–15.2) months and 7.0 (5.1–8.9) months, respectively. Thus, the development of TRAEs was associated with a significantly longer OS (*p *= 0.039).

### 3.3 Clinicopathological factors associated with the OS

The univariate and multivariate analyses of factors associated with survival after nivolumab treatment are shown in Tables 3 and 4. In the univariate analysis, macroscopic type of gastric carcinoma, histological classification, site of metastasis (liver, bone, or peritoneum), history of gastrectomy, NLR, Glasgow prognostic score, and CRP were significantly associated with the OS (Table 3). In the multivariate analysis, the histological diffuse type (HR, 2.40; 95% CI, 1.30–4.40), peritoneal metastasis (HR, 2.51; 95% CI, 1.39–4.55), and high CRP (HR, 2,89; 95% CI, 1.51–5.52) were associated with the OS (Table 4). These clinicopathological factors may predict the longer survival before nivolumab treatment.

**TABLE 3:** A univariate analysis of factors associated with the overall survival

Factor		HR	95% CI	*p* value
Gender	Male (/female)	0.83	0.48–1.43	0.491
Age	≥65 (/<65)	0.59	0.34–1.02	0.058
Macroscopic type				0.046
	2 (/0)	0.75	0.21–2.61	0.648
	3 (/0)	1.10	0.33–3.66	0.880
	4 (/0)	2.43	0.69–8.62	0.169
	5 (/0)	1.81	0.30–10.90	0.518
Histological classification	Diffuse (/intestinal)	2.50	1.44–4.33	0.001
HER2 status	Positive (/negative)	0.61	0.30–1.21	0.158
Metastatic site				
Liver	Yes (/no)	5.31	0.29–0.97	0.038
Lymph node	Yes (/no)	0.83	0.49–1.42	0.500
Bone	Yes (/no)	6.87	1.98–23.82	0.002
Lung	Yes (/no)	1.33	0.48–3.69	0.589
Peritoneum	Yes (/no)	3.00	1.73–5.20	<0.001
Other organ	Yes (/no)	1.29	0.46–3.62	0.632
History of gastrectomy	Yes (/no)	0.55	0.32–0.96	0.036
NLR	≥5 (/<5)	1.99	1.10–3.63	0.024
Glasgow prognostic score				0.019
	1 (/0)	1.32	0.70–2.49	0.386
	2 (/0)	2.82	1.35–5.90	0.006
CRP	≥1 (/<1)	3.09	1.70–5.61	<0.001
Albumin	<3.5 (/≥3.5)	1.29	0.75–2.22	0.354

Abbreviations: CRP, C‐reactive protein; NLR, neutrophil/lymphocyte ratio.

**TABLE 4:** A multivariate analysis of factors associated with the overall survival

Factor		HR	95% CI	*p* value
Histological classification	Diffuse (/intestinal)	2.40	1.30–4.49	0.005
Metastatic site				
Liver	Yes (/no)	0.56	0.28–1.11	0.098
Peritoneum	Yes (/no)	2.51	1.39–4.55	0.002
NLR	≥5 (/<5)	1.81	0.98–3.37	0.059
CRP	≥1 (/<1)	2.89	1.51–5.52	0.001

Abbreviations: CRP, C‐reactive protein; NLR, neutrophil/lymphocyte ratio.

### 3.4 The development and validation of the nomogram to predict the survival in patients receiving nivolumab treatment

**FIGURE 3:**
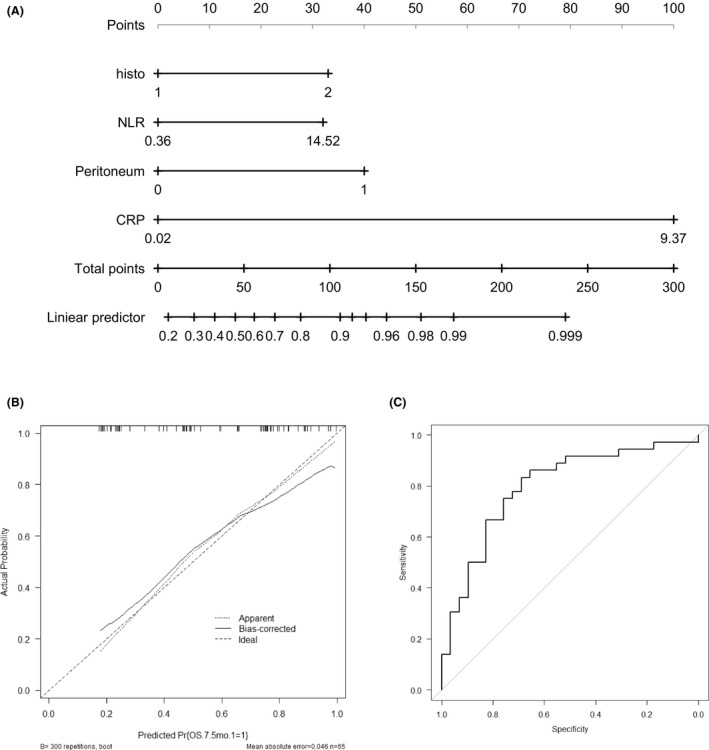
Development and validation of the nomogram. (A) The nomogram of different risk factors for survival at 7.5 months in gastric cancer patients treated with nivolumab. (B) The calibration curve of the nomogram model. (C) The receiver operator characteristic (ROC) curve of the nomogram model

Four independent risk factors (histological classification, peritoneal dissemination, NLR, and CRP) were used to develop a nomogram to predict the survival at 7.5 months after the initiation of nivolumab treatment (Figure 3A). The total points were determined as the sum of the point of the factors predicting the survival probability. An internal calibration curve was developed and the C‐index was 0.80 (95% CI, 0.68–0.91) (Figure 3B). The ROC curve indicated that area under the curve was 0.797 (95% CI, 0.685–0.910). The sensitivity was 0.750 and the specificity was 0.759 (Figure 3C).

### 3.5 Exploratory analysis

Among 13 responders, FFPE specimens were obtained from 11 cases, in which 6 were surgically resected tissue and 5 were biopsy specimens (Table S4). All were elderly patients (age ≥65 years), with the exception of one patient who was 45 years of age. The analysis of the responders included seven cases (63.6%) with the intestinal histological type. Three HER2‐positive cases and a case of gastric carcinoma with lymphoid stroma were observed. Three cases (27.3%) showed a loss of MLH1 and PMS2 antibody staining, and were classified as dMMR cancers.

## 4 DISCUSSION

A regional cohort study was conducted to acquire clinicopathological factors from a real‐world setting, and the characteristics of gastric carcinoma and biochemical data from the patients were extracted. We analyzed predictive and prognostic factors in gastric cancer patients who received nivolumab treatment. A total of 70 patients were enrolled in the cohort study in the 2 years after nivolumab for the treatment of gastric cancer received coverage by the Japanese health insurance system (in October 2017). Based on gastric cancer‐associated mortality in Japan, one quarter of patients with advanced gastric cancer received nivolumab treatment in this area. Since the characteristic data were obtained from all hospitals performing nivolumab treatment in the area, the results of this study should be widely reproducible in Japan. The median OS was 7.5 months, which was longer than that reported in the ATTRACTION‐2 trial (5.3 months) and equivalent to other retrospective studies (4.3–9.6 months).^9^, ^10^, ^11^, ^12^ The regional real‐world data supported the effectiveness of nivolumab treatment that was reported on the previous clinical trial.

The subgroup analysis showed that the mean OS of patients with a PR (responders) was 32.0 months. In the 2‐year update of the ATTRACTION‐2 trial, the median OS in patients with CR+PR was 26.61 months, while the 12‐month OS rate was 87.1%.^4^ Responders to nivolumab showed extremely long survival as a matter of course. During follow‐up among 330 patients treated with nivolumab, 3 showed a CR (1.1%) and 29 showed a PR (10.8%). Based on the analyses of the survival curves in the PR and PD cases, nivolumab was not superior to placebo. Patients with a PR showed increased survival in our cohort, with a 24‐month OS rate of 53% in these responders. Taken together, responders can obtain the long‐term survival with immunotherapy. However, the disease control rate of 41.4% was not high in comparison to the rate in patients treated with cytotoxic chemotherapy. For example, the disease control rate in patients receiving trifluridine/tipiracil treatment was 44%.^13^

Patients who had experienced AEs also survived longer in comparison to patients without AEs, as reported in a previous study.^14^ Patients with TRAEs showed a mean OS time of 10.0 months in our cohort. Although the best response and AE were associated with the OS, as previously reported, they were not predictive factors because those factors were only obtained after the initiation of nivolumab treatment. Factors that can be observed before treatment should be identified for real‐world application. Thus, the characteristics of the patients before nivolumab treatment were used in the univariate and multivariate analyses. As predictors of nivolumab efficacy for gastric cancer, both the systemic inflammatory response and nutritional biomarkers were investigated in previously published retrospective studies.^9^, ^10^ NLR was thus found to effectively predict the response to nivolumab treatment in patients with advanced gastric carcinoma.^15^ GPS, which is estimated with CRP and the albumin level which demonstrates the presence of systemic and local inflammation, has also been identified as a prognostic indicator for both survival and cancer progression.^16^ Among the characteristics of gastric cancers, the histological types had higher predictive value. A nomogram was created based on this cohort study and this trial is the first one to predict the prognosis of gastric cancer patients treated with nivolumab.

In our cohort, we found extremely long total survival from the initiation of the primary chemotherapy to the death. Patients who received nivolumab as the third‐line regime or later for gastric cancer survived >27 months. Surprisingly, the elongation of survival was associated with the development of second‐line chemotherapy or later. For example, a review reported that the second‐line chemotherapy rate influenced the OS in phase III trial series for advanced gastric cancer.^17^ Our cohort included 44 patients who received ramucirumab plus paclitaxel or nab‐paclitaxel as a second‐line treatment. The RAINBOW trial, a phase III trial of second‐line chemotherapy, showed that the OS of the patients treated with ramucirumab plus paclitaxel reached 9.6 months.^18^ Third‐line chemotherapy or later may prolong the total OS. Combination treatment with cytotoxic chemotherapy and immunotherapy may contribute to improving survival.^19^ Needless to say, our cohort included a selection bias in our patients who were received ≥third‐line chemotherapy and immunotherapy. The patients could not receive ≥third‐line chemotherapy unless their gastric cancer had responded to previous chemotherapies. Our patients frequently received TS‐1 or capecitabine plus platinum as a first‐line treatment and ramucirumab plus taxane as a second‐line treatment. These treatments are commonly used in real‐world settings in accordance with the recommendations of the guidelines.^2^, ^20^, ^21^ Our cohort was composed of patients whose tumors were sensitive to the standard chemotherapy; thus, the total survival period may have been much longer in comparison to the patients who were included in phase 3 trials.^19^

Very recently, the 3‐year update of the ATTRACTION‐2 showed that 5.6% of the nivolumab group was alive at 36 months, which is nearly half of the survival rate at 24 months (10.6%).^22^ In responders (CR+PR), 35.5% achieved survival at 36 months; in contrast, the survival rates at 24 months and 12 months were 61.3% and 87.1%, respectively. The result indicates that the survival rate decreased year‐by‐year. Any strategy should be implemented to achieve a cure. It was assumed that there are three options for obtaining longer survival. First, nivolumab treatment with another chemotherapy should be administered as the first therapy. In the ATTRACTION‐4, the combination of nivolumab with S‐1/capecitabine plus oxaliplatin showed encouraging efficacy in untreated gastric cancer patients.^23^ The median OS was not reached in this interim analysis of 1‐year follow‐up data. A future study is planned to investigate whether OS will reach to 27.5 months in our cohort. Very recently the CheckMate‐649 clinical trial compared first‐line treatment with nivolumab plus chemotherapy to chemotherapy alone, and showed that nivolumab plus chemotherapy prolonged the median OS by 3.3 months.^24^ To improve the total survival, a comparison between these combination treatments and sequential treatment with chemotherapy and immunotherapy is needed. Second, adjuvant chemotherapy with nivolumab may induce a better prognosis in patients who undergo gastrectomy. In our cohort, the patients with prior gastrectomy showed a better prognosis, as far was assessed in the univariate analysis in Table 3. Adjuvant nivolumab in resected esophageal or gastroesophageal junction cancer prolonged disease‐free survival in comparison to placebo in the CheckMate‐577 clinical trial.^24^ A further study should be performed to compare the result those of patients with resected gastric cancer. Third, biomarkers predicting the sensitivity to immune checkpoint inhibitors should be investigated. The programmed death‐ligand 1 (PD‐L1) expression was not associated with a survival benefit in the ATTRACTION‐2 trial.^1^ It is still controversial whether PD‐L1 positivity is a biomarker of sensitivity to nivolumab in gastric cancer.^25^ In a subgroup analysis of the ATTRACTION‐2 trial, in which the HER2 status was not determined, the efficacy of nivolumab was determined to be consistent in gastric cancer, regardless of prior trastuzumab use.^26^ HER2‐positive cases were also found in our cases and the HER2 expression is expected to be a marker of nivolumab sensitivity. According to The Cancer Genome Atlas (TCGA) research network, MSI and EBV infection may be predictive markers.^27^ Our limited experiments did not lead to a definite conclusions; however, same of our cases were classified as dMMR (Table S4), confirming the findings of a multicenter biomarker cohort study.^28^ Facilitating precision medicine approaches for patients with gastric cancers is proposed, with the optimized the selection of patients for targeted therapies.^29^ A nomogram based on such biomarkers or genetic phenotype is expected to predict the prognosis of gastric cancer patients who receive treatment with immune checkpoint inhibitors.

Our limitation of our cohort study was that only a small number of the patients with gastric cancer were treated with nivolumab. Only a portion of the gastric cancer patients could be treated with ≥third‐line chemotherapy. The population of Asahikawa city (~350,000) does not seem sufficient for these statistical analyses. When these analyses are conducted in a larger population, the C‐index would be expected to be higher than the result reported in this study. The nomogram should be validated in a verification cohort to confirm the clinical utility of this visual tool, which is the first nomogram constructed from gastric cancer patients treated with nivolumab.

## 5 CONCLUSIONS

A regional cohort study of nivolumab treatment for the patients with advanced gastric cancer showed median OS of 7.5 months. Among the characteristics of the patients that were assessed by a Cox regression analysis, the histological classification, site of metastasis, and CRP were found to be significant prognostic factors. A nomogram was made to identify the patients in whom the survival at 7.5 months was predicted.

## DISCLOSURE

The authors have declared no conflict of interest.

## AUTHOR CONTRIBUTIONS

K. Tan. and H.T. conceived and designed the study, analyzed the data, and drafted the manuscript. M.G., N.Y., H.A., M. Tar., S. Yo., C.I., K.H., and Y. Ki. recruited the patients and performed clinical treatment and assessments. K.S., S. Yu., and M.T. performed pathology review and histological analysis. Y. Sug., K.U., Y. Ko. Y. Mu., T.K., T.S., and S.K. performed sample recruitments. H.S. and Y. Mi. performed statistical analysis. K. Tak., K.A., and N.U. contributed to data acquisition and analysis. Y. Sum., K.M., M.F., and T.O. supervised the research and reviewed the manuscript.

## Supporting information

Table S1‐S4

## Data Availability

The data that support the findings of this study are available from the corresponding author upon reasonable request.
